# Insecticide treated eaves screens provide additional marginal protection compared to untreated eave screens under semi-field conditions in western Kenya

**DOI:** 10.5281/zenodo.10567425

**Published:** 2024-01-25

**Authors:** Bernard Abong’o, Silas Agumba, Vincent Moshi, Jacob Simwero, Jane Otima, Eric Ochomo

**Affiliations:** 1 Centre for Global Health Research, Kenya Medical Research Institute, Kisumu, Kenya; 2 Research World Limited, Kisumu, Kenya; 3 Habitat for Humanity International, Lenana Road, Nairobi

## Abstract

**Introduction:**

Human habitats remain the main point of human-vector interaction leading to malaria transmission despite the sustained use of insecticide-treated nets and indoor residual spraying. Simple structural modifications involving screening of doors, windows and eaves have great potential for reducing indoor entry of mosquitoes. Moreover, insecticide treatment of the screen material may provide additional benefit in mosquito population reduction.

**Materials and Methods:**

Four huts, each constructed inside a semi-field structure, were used in the study. Two had untreated eave and door screens and screened air cavities in place of windows (experiment 1) or were similar but with the eave screens treated with Actellic® 300CS insecticide (experiment 2). The other two huts remained unscreened throughout the study. Two hundred, 3-day old adults of F1 generation *Anopheles funestus* collected by aspiration or F0 reared from *An. arabiensis* larvae or *An. arabiensis* (Dongola strain) were released in each semi-field structure at dusk and recaptured the following morning. A single volunteer slept in each hut under an untreated bednet each night of the study. Recaptured mosquitoes were counted and recorded by location, either indoor or outdoor of each hut in the different semi-field structures.

**Results:**

Based on modelled estimates, significantly fewer, 10% *An. arabiensis* from Ahero, 11% *An. arabiensis* Dongola strain and 10% *An. funestus* from Siaya were observed inside modified huts compared to unmodified ones. Treating of eave screen material with Actellic® 300CS significantly reduced indoor numbers of *An. arabiensis* from Ahero, to nearly 0%, and *An. arabiensis* Dongola strain, to 3%, compared to huts with untreated eave screens, while eliminating *An. funestus* indoors. These modifications cost US$180 /structure and have been observed to last more than 15 years in a different location.

**Conclusions:**

Eave, door and window screening are effective ways of reducing mosquito entry into houses. Additionally, treatment of eave screen material with an effective insecticide further reduces the *Anopheles* population in and around the screened huts under semi-field conditions and could greatly complement existing vector control efforts.

## Introduction

The world recently reported an increase in estimated malaria cases in the year 2021 compared to 2020 [1]. Most of this increase in malaria cases and the greatest disease burden was reported from sub-Saharan Africa where malaria control is heavily reliant on the use of insecticide-treated bednets (ITNs), artemisinin-based combination therapy (ACT) and indoor residual spraying (IRS). ITNs were observed to have contributed the greatest to the decline in malaria transmission between the years 2000 and 2015 [2]. However, more recently, little or no progress in the reduction of malaria cases has been witnessed despite the sustained use of these interventions [1, 3].

Both ITNs and IRS are insecticide-based malaria vector control interventions whose implementations are limited to personal protection at bedtime and application on the walls respectively. Several limitations, including insecticide resistance in mosquitoes [4-7], incomplete coverage [8-10], low compliance in ITN use [11-13] and changing vector behaviour [14-17] are potentially lessening the effectiveness of these interventions. Consequently, complementary mosquito control tools are urgently required to sustain the gains made in malaria control and to further reduce the disease burden.

Malaria-transmitting mosquitoes are closely associated with human habitation. Mosquitoes are known to enter and bite within houses at night [18], thereby transmitting malaria. Even though reports of changing vector biting behaviour due to sustained ITN use exist [4, 19-21], the bulk of malaria transmission still occurs indoors in many malaria endemic settings [22, 23]. In western Kenya, recent studies have reported persistent and high numbers of late-night biting malaria vectors [24-26]. Consequently, to control malaria transmission in these regions, it is important to first identify the location of human-vector interaction to effectively target control efforts. The alteration of house designs to limit mosquito entry offers unparalleled and non-insecticide-based potential that could effectively complement malaria vector control efforts over a longer period.

Housing is a key contributor to health; it not only protects against the elements but also influences the physical and psychosocial well-being of its inhabitants. House modification has been reported in many settings to be an effective mosquito control strategy [27-30] including resulting in a positive epidemiological impact [28]. However, it has received little attention in much of sub-Saharan Africa likely due to the lack of a standardised approach and perceived cost. The World Health Organization (WHO) Global Malaria Programme recently provided a conditional recommendation for house modification based on low certainty of evidence [31]. We evaluated the impact of screening doors, windows and eaves, followed by the treatment of eave screen material with an effective insecticide in a semi-field setting to provide evidence for improved housing as a malaria control intervention and practical lessons for community implementation of a sustainable vector control intervention in western Kenya.

## Materials and Methods

**Figure 1.**
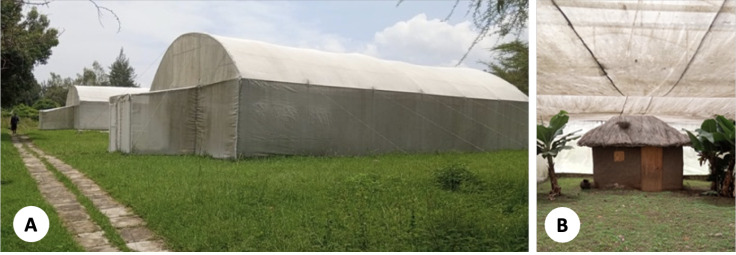
A: Semi-field structures at KEMRI-CGHR, Kisumu. B: A typical unmodified hut inside the semi-field structure.

**Figure 2.**
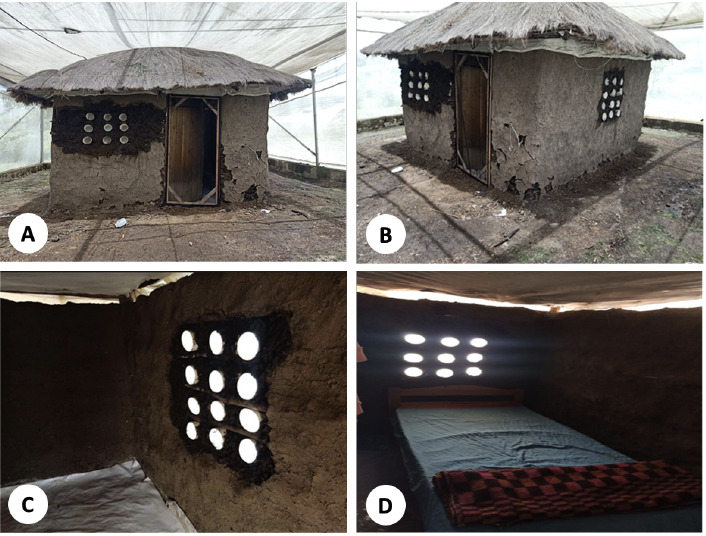
Modified huts showing front and side views with air cavities and screened doors (A and B), interior views of the living room (C) and bedroom (D) areas.

The experiments were conducted at the Kenya Medical Research Institute – Centre for Global Health Research (KEMRI – CGHR) in Kisumu, western Kenya. The campus has four semi-field stations which are double-netted, double-door structures measuring 20 m in length and 8 m wide and rise to 4.5 m at the apex [32, 33]. We modified the semi-field structures with a 3 m high netting to ensure ease of mosquito recapture. Each semi-field structure houses one hut measuring 3 m (L) by 3 m (W) by 2 m (H) (Figure 1). Each hut has an open ceiling, a wooden door and two windows and are similar to a typical simple house structure in the western Kenya region (Figure 2).

### Modification of the structures

Figures 2A-D show structural modification of the huts involving screened eaves, door and air cavities. Structural modifications were performed by replacing windows with screened air cavities and screening doors and eaves. The replacement of windows with screened air cavities was based on a proposal to introduce the air cavities in houses that have no or very small windows to enable light and air flow in the house while preventing mosquito entry. The modifications were performed only for the huts in screenhouse 2 and 3 while screenhouse 1 and 4 served as controls. The air cavities were made of PVC pipes of 20 cm diameter and 15 cm length. These were fitted with insect mesh (16 to 18 holes per square inch) to allow air and light while blocking the passage of mosquitoes and were arranged into a panel of nine or twelve depending on the size of the window space. The gaps between the pipes were filled with mud with the same material as used for walls. Eaves were screened by attaching an insect mesh (16 to 18 holes per square inch) to the edge of the wall on one side and to the rafters and purlins on the roof on the other end to cover the eave space. The door to each hut was screened by introducing an openable wood-framed screen shutter to the outside of the main door. An insect screen mesh was attached to the wood-framed shutter using Velcro. For experiment 1, untreated insect screen material was used to screen the eaves whereas, in experiment 2, the insect mesh was treated with pirimiphos methyl (Actellic® 300CS).

### Mosquito collections

Wild *Anopheles arabiensis* larvae were collected from rice fields in Ahero, Kisumu County and reared to to 3-day-old adults at the KEMRI-CGHR insectaries in Kisumu. Adult wild *An. funestus* were collected by Prokopack aspiration from Uranga in Siaya County, western Kenya and transported to KEMRICGHR insectaries for rearing of the F1 generation. Gravid female *An. funestus* were provided with a laying pad inside a cage to collect eggs. Once eggs had hatched, the resulting larvae were reared under insectary conditions (27±2°C, 80±10% RH) to 3-day-old adults for the release experiments. Laboratory-reared, 3-day-old adult *An. arabiensis* (Dongola strain) was included in the release experiments. *Anopheles funestus* has high intensity (10X) resistance to pyrethroids while *An. arabiensis* from Ahero has low-intensity (2X) resistance to the same class of insecticides. The *An. arabiensis* Dongola colony is susceptible to all insecticides. Based on results from a separate experiment with the same mosquito strains, all experimental mosquitoes were fully susceptible to organophosphates (Agumba *et al.*, in prep.).

### Semi-field experiments

Two experiments were conducted. In experiment 1, the eaves of the modified huts were screened with untreated insect mesh material. In this experiment, 5 releases of *An. arabiensis* from Ahero, 2 releases of *An. arabiensis* Dongola strain and 4 releases of *An. funestus* F1 generation from Siaya were done in the semi-field structures over a total of 11 nights in March 2022. Each release comprised 200 female mosquitoes per semi-field structure.

In experiment 2, eave screens were treated with Actellic® 300CS (pirimiphos-methyl). For this trial, 833 ml of Actellic® 300CS was diluted in 6.67 litres of tapwater in a 15 L capacity H. D. Hudson Manufacturing Company (Chicago, IL) 67422 AD, Hudson X-pert spray pump recommended by WHO for use in IRS. The mixture was pressurised to 55 psi [34]. The application rate was 1 m in 2.2 s for 2 m^2^ area of netting [35]. Netting material (4x1 m) was attached to a board, sprayed and allowed to dry in the shade following WHO guidelines [35]. It was wrapped in airtight polyethylene and kept at room temperature. In experiment 2, 5 releases of each of the three mosquito strains were performed over 15 nights in June 2022. Each release comprised 200 female mosquitoes per semi-field structure.

During each experiment, an adult male volunteer was recruited and slept under an untreated net in each of the huts. The volunteers were required to stay inside the huts from 20.00 hrs until 06.00 hrs the following morning except for restroom breaks. Mosquitoes were released between 18.00 and 19.00 hrs each evening from the centre of each semi-field structure. Collection of the released mosquitoes was done in two sets, the first collection between 06.00-07.00 hrs and a second and final collection between 09.00-10.00 hrs the next day. Mosquito collection was done using mouth aspiration as well as mechanical aspiration using Prokopack aspirators Model 1419, John W. Hock Company. Mosquitoes collected indoors or outdoors were kept in separate cups and labelled by the semi-field structure number as well as the location of capture for counting.

### Data analysis

Recaptured mosquitoes were counted, and numbers recorded by trapping location as either indoors or outdoors of each hut. For analysis, data were entered into Excel and imported into R statistical software version 4.1.2. The risk ratio (RR) was used to assess the statistical significance of differences in mosquito numbers between screened and unscreened huts. The over-dispersed data were fitted using a Generalised Linear Mixed Model using Template Model Builder (glmmTMB)' with a negative binomial distribution for the analysis of mosquito numbers between screened and unscreened huts. The total number of each Anopheles strain recaptured indoors and outdoors of each hut was assessed as a fixed effect function of intervention status (screened or unscreened) while hut and sleeper were considered random effects. To obtain the risk ratios (RR) and confidence intervals, we exponentiated the model coefficients.

## Results

In both experiments the highest numbers of mosquitoes recaptured indoors were from unscreened huts compared to modified ones. The number of mosquitoes of all strains was relatively higher outdoors in semi-field structures with screened huts compared to those with unscreened huts. Recapture rates were highest for *An. arabiensis* Dongola strain, followed by wild *An. funestus* from Siaya and lastly the wild *An. arabiensis* from Ahero in both experiments 1 and 2 (Table 1).

**Table 1.** Numbers of mosquitoes recaptured indoors and outdoors of screened and unscreened huts within the semi-field structures for both experiments (1 & 2).

Experiment #	Location & species	Treatment	Indoor	Outdoor	Number released	Number recaptured	% Recaptured
1	Ahero (*An. arabiensis*)	Hut1_Unscreened	109	85	1000	194	19.4
		Hut2_Screened	17	191	1000	208	20.8
		Hut3_Screened	1	185	1000	186	18.6
		Hut4_Unscreened	35	156	1000	191	19.1
	Dongola (*An.* *arabiensis*)	Hut1_Unscreened	120	53	400	173	43.3
		Hut2_Screened	15	213	400	228	57.0
		Hut3_Screened	6	231	400	237	59.3
		Hut4_Unscreened	80	188	400	268	67.0
	Siaya (*An.* *funestus*)	Hut1_Unscreened	117	93	800	210	26.3
		Hut2_Screened	18	209	800	227	28.4
		Hut3_Screened	8	229	800	237	29.6
		Hut4_Unscreened	135	85	800	220	27.5
2	Ahero (*An.* *arabiensis*)	Hut1_Unscreened	140	165	1000	305	30.5
		Hut2_Screened	1	139	1000	140	14.0
		Hut3_Screened	0	150	1000	150	15.0
		Hut4_Unscreened	188	144	1000	332	33.2
	Dongola (*An.* *arabiensis*)	Hut1_Unscreened	354	124	1000	478	47.8
		Hut2_Screened	6	295	1000	301	30.1
		Hut3_Screened	12	333	1000	345	34.5
		Hut4_Unscreened	294	158	1000	452	45.2
	Siaya (*An.* *funestus*)	Hut1_Unscreened	147	140	1000	287	28.7
		Hut2_Screened	0	183	1000	183	18.3
		Hut3_Screened	0	204	1000	204	20.4
		Hut4_Unscreened	133	188	1000	321	32.1
Total			1936	4141	20800	6077	29.2

### Experiment 1

**Figure 3.**
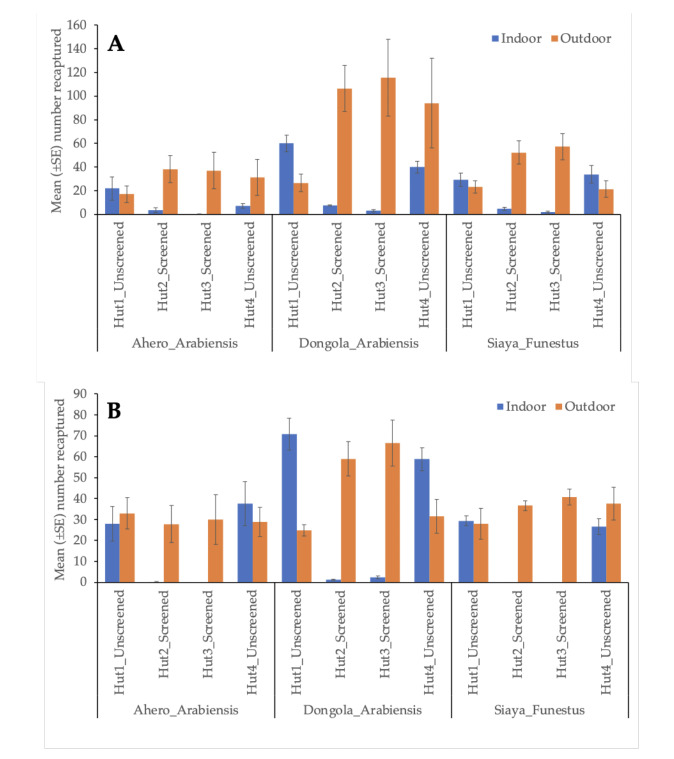
A: The mean number (±SE) of *An. arabiensis* from Ahero, *An. arabiensis* Dongola strain and *An. funestus* from Siaya recaptured indoor and outdoor screened and unscreened huts within the semi-field structures. Huts 1 and 4 were unscreened while Huts 2 and 3 were screened. B: As above, except that the

The mean number of each mosquito strain recaptured indoors and outdoors of screened and unscreened huts are presented in Figure 3A. Relatively higher numbers of *An. arabiensis* from Ahero, *An. arabiensis* Dongola strain and *An. funestus* from Siaya were observed outdoors of screened huts 2 and 3 compared to the numbers indoors. For unscreened huts, no difference in indoor and outdoor numbers of *An. arabiensis* from Ahero were observed in hut 1, except for hut 4 where relatively more were collected outdoors. Higher numbers of *An. arabiensis* Dongola strain were recaptured indoors in unscreened hut 1 whereas higher numbers were recaptured outdoors of hut 4. The numbers of *An. funestus* from Siaya were slightly higher indoors compared to outdoors, but the differences were not significant (Figure 3A).

### Experiment 2

Relatively higher numbers of all the mosquito strains tested were recaptured outdoors compared to indoors for all screened huts 2 and 3. No difference in the numbers of *An. arabiensis* from Ahero were observed between indoor and outdoor of unscreened huts 1 and 4. For *An. arabiensis* Dongola Strain, relatively more were recaptured indoors compared to the outdoors of unscreened huts 1 and 4. The numbers of *An. funestus* from Siaya were not different between indoors and outdoors of the unscreened huts 1 and 4 (Figure 3B).

In experiment 1, significantly fewer *An. arabiensis* from Ahero [RR=0.10; (95%CI: 0.02-0.63); P=0.0145], *An. arabiensis* Dongola strain [RR=0.11; (95%CI: 0.04 – 0.19); P<0.0001 and *An. funestus* from Siaya [RR=0.10; (95%CI: 0.06-0.17); P<0.0001] were observed inside modified huts compared to unmodified ones. For outdoor collections, no significant difference was observed in the numbers of *An. arabiensis* from Ahero [RR=1.56; (95%CI: 0.72-3.38); P=0.259] and *An. arabiensis* Dongola strain [RR=2.10; (95%CI: 0.87-5.05); P=0.0985] between modified and unmodified huts. However, there were significantly more *An. funestus* from Siaya outside modified huts compared to unmodified ones [RR=2.46; (95%CI: 1.61-3.77); P<0.0001] (Table 2).

**Table 2.** Mean number caught indoors or outdoors per species/strain for screened and unscreened huts, including Risk Ratios (RR, with 95% confidence intervals).

Experiment #	Species/Strain	Recapture location	Treatment	Mean	RR (95% CI)	P value
Experiment 1 (Untreated eave screens)	*An. arabiensis* Ahero	Indoors	Screened	1.80	0.10(0.02-0.63)	0.01 45
			Unscreened	14.40	Ref	
		outdoors	Screened	37.60	1.56(0.72-3.38)	0.2 59
			Unscreened	24.10	Ref	
	*An. arabiensis* Dongola	Indoors	Screened	5.25	0.11(0.04-0.19)	<0.0001
			Unscreened	50.00	Ref	
		Outdoors	Screened	111.00	2.10(0.87-5.05)	0.09 85
			Unscreened	60.25	Ref	
	*An. funestus* Siaya	Indoors	Screened	3.25	0.10(0.06-0.17)	<0.0001
			Unscreened	31.50	Ref	
		Outdoors	Screened	54.75	2.46(1.61-3.77)	<0.0001
			Unscreened	22.25	Ref	
Experiment 2 *(treated eave screens)*	*An. arabiensis* Ahero	Indoors	Screened	0.10	0.003(0.00 - 0.03)	<0.0001
			Unscreened	32.80	Ref	
		Outdoors	Screened	28.90	0.94(0.50-1.75)	0.83
			Unscreened	30.90	Ref	
	*An. arabiensis* Dongola	Indoors	Screened	1.80	0.03(0.02-0.05)	<0.0001
			Unscreened	64.80	Ref	
		Outdoors	Screened	62.80	2.23(1.61-3.08)	<0.0001
			Unscreened	28.20	Ref	
	*An. funestus* Siaya	Indoors	Screened	0.00	-	-
			Unscreened	28.00	Ref	
		Outdoors	Screened	38.70	1.18(0.87-1.60)	0.285
			Unscreened	32.80	Ref	

In experiment 2, huts with treated eave screen material yielded significantly fewer *An. arabiensis* from Ahero [RR=00; (95%CI: 0.00-0.03); P<0.0001] and *An. a r abi ens is* Dongol a strain [RR=0.03; (95%CI:0.02-0.05); P<0.0001] indoors compared unscreened ones. Hut screening with treated eave material eliminated the occurrence of *An. funestus* indoors. Significantly higher numbers of *An. arabiensis* Dongola strain were recaptured outdoors of screened huts compared to unscreened ones [RR=2.23; (95%CI: 1.61-3.08); P<0.0001]. No significant differences were observed for *An. arabiensis* from Ahero and *An. funestus* from Siaya outdoors in screened compared to unscreened huts (Table 2).

The densities of *An. arabiensis* from Ahero, *An. arabiensis* Dongola strain and *An. funestus* from Siaya recaptured inside and outside screened huts with untreated eave screens (Experiment 1) were compared to screened huts with treated eave screens (Table 3). Significantly fewer *An. arabiensis* from Ahero [RR=0.05; (95%CI: 0.00-0.77); P=0.0311] and *An. arabiensis* Dongola strain [RR=0.34; (95%CI: 0.18-0.64); P=0.000861] were recaptured indoors in huts with treated eave screens compared to huts with untreated eave screens. No *An. funestus* were recaptured inside huts with treated eave screens. In outdoor collections, significantly fewer *An. arabiensis* Dongola strain [RR=0.57; (95%CI: 0.41-0.78); P=0.000615] and *An. funestus* from Siaya [RR=0.71; (95%CI: 0.55-0.91); P=0.00607] were recaptured outside huts with treated eave screens compared to huts with untreated eave screens.

**Table 3.** Comparison of mean number of *An. arabiensis* from Ahero, *An. arabiensis* Dongola strain and *An. funestus* from Siaya recaptured indoors and outdoors between screened huts with untreated eave screens (Experiment 1) and screened huts with Actellic® 300CS-treated eave screens (Experiment 2).

Species/strain	Recapture location	Experiment	Mean	RR (95% CI)	P value
*An. arabiensis* Ahero	Indoor	2	0.10	0.05(0.00-0.77)	0.0311
		1	1.80	Ref	
	Outdoor	2	28.90	0.77(0.39-1.52)	0.449
		1	37.60	Ref	
*An. arabiensis* Dongola	Indoor	2	1.80	0.34(0.18-0.64)	0.000861
		1	5.25	Ref	
	Outdoor	2	62.80	0.57(0.41-0.78)	0.000615
		1	111.0	Ref	
*An. funestus* Siaya	Indoor	2	0.00	-	-
		1	3.25	Ref	
	Outdoor	2	38.70	0.71(0.55-0.91)	0.00607
		1	54.75	Ref	

The cost of modification of the huts under semi-field conditions were estimated at $180 per hut. This included the installation of a screen door and eave screens in addition to two sets of air cavities, one with 9 and another with 12 air cavities per house (Table 4).

**Table 4.** Costs of installation of screened air cavities, eave and door screening per structure under semi-fied conditions in western Kenya. The exchange rate at the point of modification was $1 = KES 115.54.

Estimated parameter	Description	Total cost (KES)	Total cost (US$)
Materials	Insect screen	8,400	72,73
	Door hinges	200	1,73
	Insect wire	1,000	8,66
	Timber	1,250	10,82
	Tack nails	400	3,46
	PVC Pipes	3,000	25,97
	Nails	540	4,68
Labour		6,000	51,95
Total		20,790	180

## Discussion

Screening of doors, windows, and eaves significantly reduced indoor entry of Anopheles mosquitoes into huts under semi-field conditions. Treating the eave screen material with Actellic 300CS was observed to further reduce the numbers of *An. arabiensis* from Ahero and Dongola strains inside screened huts while eliminating the occurrence of *An. funestus* indoors. Additionally, screening was observed to prevent entry into the huts thus the increase in numbers of mosquitoes recaptured outside the huts, whereas insecticide treatment of eave screen material significantly reduced the numbers of *An. arabiensis* Dongola strain and *An. funestus* outdoors, within the semi-field structures. Treated eave screens thus become a killing agent as evidenced by reduced numbers of mosquitoes recaptured indoors and outdoors of those huts. Whereas house screening provides a physical barrier that limits entry of mosquitoes into the huts, addition of insecticides in the screen material provide additional marginal benefit in reducing the vector population by killing them.

House screening has demonstrated potential for controlling disease-transmitting vectors such as mosquitoes [27,29,36-38]. Mosquitoes are adapted to enter and feed within houses [18], hence transmitting malaria. Eaves are the main avenues for house entry by mosquitoes [18,39], with doors and windows being additional routes. Blocking of eaves has been demonstrated to significantly reduce the number of mosquitoes indoors [29,40] while additional screening of doors and windows increases the success of reducing mosquitoes indoors [36,41]. In western Kenya, the major malaria vectors bite indoors, late in the evening and at night [24-26] despite sustained use of ITNs. House screening therefore offers practical options for mosquito control in the region. Consistent with previous studies in the region, we observed inhibition of entry of local *An. arabiensis* from Ahero and *An. funestus* from Siaya into huts under semi-field conditions.

Screening of huts was observed to limit the entry of mosquitoes indoors while increasing the numbers recaptured outdoors within the semi-field structure. Similar observations are likely to be made under actual field conditions. Malaria vectors are usually closely connected with human dwellings [42], mostly occurring within the peri-domestic space, either indoors or outdoors. One inadvertent result of house screening could be the increase in outdoor malaria transmission which remains poorly understood and controlled. This challenge has been previously observed with the implementation of ITNs, where increased coverage and use of ITNs have been associated with elevated outdoor malaria transmission [14,16,17,43,44]. Insect-proof housing is more likely to exacerbate the already challenging outdoor malaria transmission, therefore vector population reduction options need to be explored in combination with house screening.

Treating screening material with an effective insecticide presents an option for vector population reduction. We observed the treatment of eave screen material to significantly reduce the numbers of *An. arabiensis* Dongola strain both indoors and outdoors, *An. arabiensis* Ahero strain indoor and *An. funestus* outdoors while eliminating the numbers indoors under semi-field conditions. Anopheles mosquitoes mostly enter and exit houses through the eaves [18], therefore, treating eave screens offers a viable option for the reduction of mosquito populations. Consistent with these observations, eave tubes treated with pyrethroids have been demonstrated to be effective in preventing house entry and reducing mosquito populations under semi-field conditions [45] and in the field [46]. A combination of house screening with the delivery of insecticides on the eaves screen material offers greater protection against endophilic and endophagic mosquitoes. However, care should be taken in the choice of insecticides to ensure cost effectiveness and efficacy, and mitigate against the spread of insecticide resistance in mosquitoes.

The different strains of malaria vector species used in this evaluation were affected differently by house screening and treatment of eave screen material. Screening significantly reduced the numbers of *An. arabiensis* from Ahero indoors, however, there was no significant difference in the numbers outdoors between screened and unscreened huts. Similar results were observed when the eave screen was treated with Actellic 300 CS. Furthermore, the treatment of eave screens only reduced the numbers indoors but did not have an impact on the numbers outdoors. These observations are consistent with the reported behaviour of *An. arabiensis* in western Kenya. The species has been reported to be more exophagic and exophilic [47,48]. Consequently, the numbers observed in this study to have entered the unscreened huts were not many such that no difference was observed in the numbers outdoors between the two treatment groups.

*An. arabiensis* Dongola strain exhibited the highest recapture rate both indoors and outdoors compared to the field-caught species. Screening significantly reduced indoor entry of the species and further reductions were observed with treated eave screen material. The response of *An. arabiensis* Dongola strain to house screening may, however, not reflect the actual field situation since the species used is a laboratory colony and may have lost some of its natural traits.

House screening resulted in significant reductions in the numbers of *An. funestus* indoors while increasing the numbers outdoors. Treating eaves screen material with Actellic 300CS eliminated the species indoors while significantly reducing the numbers outdoors when compared with untreated eaves screen material. *An. funestus* in western Kenya has been reported to be highly endophilic and endophagic [47, 48] hence feeding and resting more frequently indoors. The species is therefore more inclined to indoor entry. Consistent with these reports, we observed screened huts to have significantly lower numbers indoors compared to unscreened huts, whereas the numbers outdoors of screened huts were higher than those around unscreened huts. Treatment of eave screen material further affected the population of *An. funestus* indoors and outdoors of the huts. Previous studies in the region have reported the species to be highly susceptible to an effective insecticide. *An. funestus* was reduced to near elimination in the Asembo Bay area with the introduction of ITNs [49]. Similar results were observed in Migori County, western Kenya, following a single indoor residual spray (IRS) campaign with Actellic 300CS [26].

With screened eaves, doors and windows, it is anticipated that no mosquitoes would gain entry into the huts. However, mosquitoes were recaptured inside screened huts albeit in low numbers compared to unscreened ones. This is evidence that the huts were still porous to mosquito entry and the doors may have provided passage of mosquitoes indoors as volunteers moved into and out of the huts. A study assessing the impact of screened doors in excluding mosquitoes from houses in rural Gambia observed numbers of mosquitoes to increase indoors with increased frequency of door opening at night [41]. Doors are therefore the major point of weakness which requires special consideration in house screening for mosquito control.

The cost of modifying a single hut was estimated at US$180. Other vector control tools such as LLINs are costed at approximately US$2.00 [50]. Comparatively speaking, the initial cost for house modification is therefore considerable. However, it is cost effective over time because a single modification would possibly last the entire lifetime of the house [29], while providing protection to all occupants indoors. In western Kenya, traditional mud walled, grass thatched houses are reported to last for a generation, which is 20-30 years, before being demolished [51]. However, such houses undergo routine maintenance with repair of walls, roofs and floors. With such routine maintenance by house-owners, housing modifications for vector proofing are likely to last longer and become more cost effective.
